# Comparison of a DNA Based PCR Approach with Conventional Methods for the Detection of *Mycobacterium tuberculosis* in Morocco

**DOI:** 10.4084/MJHID.2012.049

**Published:** 2012-08-09

**Authors:** Fathiah Zakham, Oufae Lahlou, Mohammed Akrim, Nada Bouklata, Sanae Jaouhari, Khalid Sadki, Fouad Seghrouchni, Mohammed Elmzibri, Abdelaziz Benjouad, Mustapha Ennaji, Rajae Elaouad

**Affiliations:** 1 Institut National d’Hygiène, Rabat, Morocco.; 2 Laboratoire de Virologie et Hygiène & Microbiologie, Faculté des Sciences et Techniques. Mohammedia, Morocco.; 3 Laboratoire de Biochimie et Immunologie, Faculté des Sciences. Université Mohammed V-Agdal. Rabat. Morocco.; 4 Unité de Biologie et Recherches Médicales, Centre National de l’Energie, des Sciences et Techniques Nucléaires-Rabat-Morocco.

**Keywords:** Tuberculosis, Mycobacterium tuberculosis, PCR, IS6110

## Abstract

**Background:**

Worldwide, tuberculosis (TB) is a major public health problem and the rapid diagnosis and appropriate chemotherapy become the first priority and a serious challenge to improve TB treatment. In the objective of early TB diagnosis and rapid detection of Mycobacterium tuberculosis (MTB) in the clinical specimens, the utility of the Polymerase Chain Reaction (PCR) using the Insertion Sequence 6110 “IS6110" as target was compared to conventional methods.

**Methods:**

Out of 305 patients with different clinical manifestations: suspected, new, drug relapse, drug failure and chronic cases were enrolled in this study and tested by mycobacteriological and PCR techniques for the investigation about the tubercle bacilli.

**Results:**

The results of the in house “IS6110" PCR showed a good sensitivity (92.4%) and high specificity (98.0%), the positive and negative predictive values were 96.4 % and 95.3 % respectively.

**Conclusion:**

This study showed clearly that the PCR testing using the “IS6110" in the routine analysis is a potential tool for the rapid TB diagnosis, especially for critical cases and would be of great interest to help the clinician in the misdiagnosed critical cases by the traditional radiology.

## Introduction

*Mycobacterium tuberculosis* (MTB) is the causative agent of Tuberculosis (TB), which is responsible for 8 to 10 million new cases of TB and 2 million deaths annually.^1^ In Morocco, TB is a major problem of the public health with a high incidence reaching 82.1 new cases for 100 000 inhabitants.^2^ Tuberculosis affects especially young adults and therefore has a high impact on the socio-economic status of the country.

The early and rapid diagnosis of MTB is very important for controlling and initiation drug treatment regimen. The laboratory diagnosis of TB is mainly based on the microscopic examination by the Ziehl-Neelsen staining and MTB culture, which are widely used in the laboratories of public health centres for the routine analysis.^3^,^4^ Ziehl-Neelsen staining is a cheap technique and easy to perform but lacks sensitivity and is unable to distinguish between MTB members and other atypical mycobacteria. Moreover, Ziehl-Neelsen staining can only detect acid-fast bacilli in concentrations exceeding 10,000 organisms per ml.^5^ MTB culture is the gold standard method but it requires viable microorganisms and long time incubation (up to 4 weeks), representing a problem especially for patients with critical situations such as immunocompromised or AIDS patients.

To overcome these limitations, molecular approaches have been introduced into clinical mycobacteriology laboratories. In this field, the most common technique used is the DNA amplification by the Polymerase Chain Reaction (PCR). The opportunity to use PCR for the detection of MTB in clinical samples has been reported.^7^–^9^ Many MTB DNA sequences; including 16S rDNA and hsp65 genes and “IS6110" are used as targets for MTB detection by PCR.^10^–^12^

The insertion sequence “IS6110" is a transposable element which is present in the members of MTB complex in multiple copies (up to 25 copies), except *M. bovis* BCG which harbours a single copy and absent in other mycobacteria.^13^,^14^ For those reasons the insertion sequence “IS6110” has been reported as the most common target used for the MTB diagnosis in the clinical samples and demonstrated that the detection rate of MTB complex targeting “IS6110” was higher than that of microscopy or MTB culturing with a considerable time.^7^,^12^,^15^,^16^ Furthermore, due to its high numerical and positional polymorphism, “IS6110" sequence has become a widely used marker in the epidemiological studies^17^,^18^ and the fingerprinting of this transposable element has been used for the strain identification^19^ and phylogenetic analysis.^14^ Consequently, “IS6110" sequence is a useful and reliable tool for the diagnosis of mycobacterial strains in clinical specimens.^20^–^22^ Thus, this study was planned to evaluate the use of PCR for “IS6110" sequence amplification for rapid diagnosis of MTB in clinical samples to improve the TB management in Morocco.

## Materials and Methods

### Sampling

A total of 305 specimens were collected from different hospitals in and around Rabat city and sent to the National laboratory of Reference in Tuberculosis (LNRT) at the National Institute of Hygiene in Rabat for MTB detection from February 2010 to May 2011.

Patients have been classified to five groups according to their clinical manifestations: suspected, new, treatment failure, treatment relapse and chronic TB cases depending on the symptoms and radiological examination. The suspected cases were sent to LNRT for an initial assessment of TB. The new cases were sent to confirm the infection with MTB, without undergoing to any drug treatment regimen.

Patients suffering of drug failure or relapse and chronic cases don’t show any improvement in their health even with treatment after six months and they are still having TB profile in their radiological examination.

Most of the specimens were pulmonary (sputum, sputum induced by fibre-optic bronchoscopy, bronchial wash and bronchial aspirations), and some of them were extrapulmonary (pleural fluid, abscess, urine and gastric liquid).

All the pulmonary specimens were decontaminated and liquefied with N-acetyl-l-cysteine (NALC) method,^23^ the biopsy was minced, homogenized in a sterile homogenizer and concentrated. The rest of specimens were concentrated by centrifugation as mentioned by Ben Kahla et al,^24^ the inoculation was performed on Lowenstein Jensen (L/J). A part of each decontaminated specimen was sent to the Molecular biology laboratory for MTB detection by PCR.

### Bacterial lysis

Bacterial lysis was performed as described by Aldous, *et al*.^25^ Briefly, the specimen was first thawed and centrifuged at 6,000 *g* for 1 min. The supernatant was discarded and the pellet was resuspended in 200 μl of TE buffer and processed in boiling water bath for 15 min to inactivate bacteria and release DNA. After centrifugation at 16,000 *g* for 5 min, aliquot of 100 μl of the supernatant was transferred to a sterile tube. DNA was immediately used for PCR amplification or stored at −20°C until use.

### PCR for amplification of the insertion sequence IS 6110

The primers 5′-CCTGCGAGCGTAGGCGTCGG-3′ and 5′-CTCGTCCAGCGCCGCITCGG-3′, were used to amplify a 123-base-pair fragment as described by Eisenach, *et al*.^20^ The amplification reaction was performed in a total volume of 50 μl of the amplification mixture contained 0.2 μM of each primer, 0.2 mM of each dNTPs, 2.5 mM MgCl, 5 Units of Taq DNA polymerase enzyme (Roche diagnostic, GmbH, Manheim, Germany) and 3 μl of DNA sample in 1x *Taq* polymerase buffer.

PCR reactions were performed in thermal cycler (Gene Amp, PCR system 9700, Applied Bio system). The mixture was first denatured at 95°C for 10 min. Then, 40 cycles of PCR were performed with denaturation at 95°C for 1 min, primer annealing for 1 min at 68°C and primer extension for 1 min at 72°C. At the end of the last cycle, the mixture was incubated at 72°C for 10 min. For every reaction a negative control, in which DNA template was omitted from the amplification mixture, and a positive control, containing DNA from H37Rv MTB strain are included. PCR products were analysed by electrophoresis on 2% agarose gels followed by staining with ethidium bromide (10 mg/ml).

### Statistical analysis

Specificity, sensitivity, positive and negative predictive values were calculated were calculated in combination with the bacterial culture, as a gold standard technique.^26^ For testing the agreement between culture and molecular approach, the kappa index was applied.^27^

## Results

A total of 305 specimens were enrolled in this study. The demographical and epidemiological information about the patients were assessed by a questionnaire regarding (range of age, age median, percentage of each sex and sex ratio) are summarized in Table 1 and showed that the age medians of the different groups were forties with a significant male predilection. The results of mycobacteriological examination and PCR testing according to their clinical manifestation are also outlined in Table 1. Among the305 specimens, positive results were obtained in 75, 116 and 111 cases by direct examination, culture and PCR respectively.

The results showed that culture is more sensitive than the direct examination in detection of MTB in all groups of cases. Furthermore, the results of conventional examination and Molecular testing according to the kind of specimens are summarized in Table 2.

Comparison of results obtained by direct examination with the molecular approach is reported in Table 3 and showed a significant difference. In fact, for 43 smear negative cases, the presence of MTB DNA was revealed by PCR.

Seven smear positive with low number of seen acid fast bacilli AFB were negative by PCR approach. Moreover, among the 305 specimens, 187 were negative by Ziehl Nielsen and PCR, and 68 were positive by direct examination and confirmed by PCR testing.

Comparison of MTB detection in the 305 specimens by both culture and PCR is reported in Table 4. Similar results were obtained for 292 clinical specimens. A total of 107 cases were positive by both MTB culture and PCR testing, and 185 cases were MTB negative by culture and confirmed by PCR. However, discordance was obtained for 13 cases. A total of 9 samples were positive in culture and negative in PCR. Four samples were positive by PCR and negative in culture; with strong sever symptoms of clinical manifestations of TB as reported in the clinical data. Among these 4 samples, two were positive in the direct microscopic examination but in low numbers of acid fast bacilli (AFB).

Depending on these results, specificity and sensitivity of the direct PCR compared to the culture as “gold standard” were calculated of the 305 clinical samples. Therefore, the PCR technique has a good sensitivity (92.6 %) and high specificity (98.0 %). The positive and negative predictive values were 96.4 % and 95.3 % respectively.

Moreover, the statistical test for the accordance between PCR and culture was expressed by kappa index with an excellent agreement of 0.9088.

## Discussion

The diagnosis of tuberculosis poses a major problem and a serious challenge, especially in countries with limited resource, including Morocco. On the international scale, the TB management remains a nightmare and the early diagnosis of MTB and drug resistance testing confront a great defy and a high challenge, particularly in the paucibacillary specimens such smear negative sputum, biopsies, pus and body fluids.^28^ Classical radiography still the first step of diagnosis after the suspicion of TB by the clinical manifestation, but it is not specific for the detection of MTB, and merely the identification of the microorganism in the specimens can confirm the diagnosis.^4^ Most of people in critical cases are usually elder, aged between 48 to 80 years and probably suffering from other underlying diseases rather than TB and can show atypical clinical presentation of TB.^3^ Furthermore, it was unexpected to misdiagnose a lot of cases as chronic, drug relapse or drug failure according to their clinical history.

Currently, the laboratory diagnosis of TB is based on the conventional techniques, including the method of Ziehl-Neelsen staining which lacks sensitivity and MTB culture that is time consuming.^5^,^6^ In this study, the MTB culture provided high sensitivity in comparison with the direct examination, 116 in culture versus 75 with smear microscopy. These results are in agreement with the worldwide use of MTB culture as a “gold standard” technique.^3^

The implementation of reliable and rapid molecular techniques for the detection of TB strains is likely to be necessary to improve health surveillance of TB. Therefore, this study was done to evaluate the utility of the molecular approach based on PCR testing to detect MTB DNA directly from clinical specimens. Many studies have evaluated the detection of limited mycobacteria cells, from clinical specimens, by PCR technique targeting different sequences such as 16S rDNA and hsp65 genes, and “IS6110". The 16S rDNA gene is one of the most used genes for this purpose,^10^ but its utilisation is limited by the need of PCR product sequencing. Moreover, PCR targeting 16S rDNA gene can not differentiate between the closely related species of mycobacteria.^10^ The *hsp65* was also reported to be used as a target for PCR testing to detect MTB DNA in clinical cases, but the necessity of sequencing is still mandatory for the differentiation between the complex MTB and other non tuberculosis mycobacteria.^11^,^29^

The insertion sequence “IS6110" which exists only in MTB complex members, is the most used target for the mycobacterial DNA amplification^7^,^12^,^20^ and is the standard marker for the epidemiologic studies of TB. Moreover, the “IS6110" is a potential tool to differentiate between the members of MTB complex and other non tuberculosis and atypical mycobacteria.^18^,^21^,^30^ In this context, it is noteworthy to mention that the IS1081 has also been used for the detection of MTB complex in the paucibacillary specimens.^31^

The results obtained in each group of patients according to their TB profile do not show a significant difference between culture and PCR. In fact, TB profile depends especially on the virulence of the bacteria and the immunity of the patient, and could be a consequence of drug failure or abandon of treatment. But, bacterial culture and PCR testing rest on the presence of viable cells and bacterial DNA respectively.

The corner stone of this study is the rapid detection of MTB in the clinical specimens by molecular approach among critical MTB cases. Thus, Among the 305 tested cases, 107 strains were detected by PCR with high positive and negative predictive values. When comparing the results obtained by PCR with the direct microscopic examination, a wide gap was found (75 detected by the direct examination versus 111 positive by PCR). The 7 positive smears and negative by PCR were recorded with low numbers of AFB and were also negative by culture. Indeed, the culture requires viable microorganisms and this is a huge problem especially in people under chemotherapy. Moreover, it’s widely accepted that the detection limit of PCR is generally 10 mycobacterial genome copies; the sputum samples with a score of AFB 1+ may contain smaller amounts of required DNA to be amplified.^32^ On the other hand, MTB DNA from 43 specimens smear negative was detected by PCR, giving a clear evidence of the utility of PCR testing to enhance direct detection of MTB.

The comparison of PCR testing to the “Gold standard” technique gives a high sensitivity (98%) and good specificity (92.6 %) and the agreement between PCR and culture was 0.9088, which is an excellent value. The specificity and sensitivity of detection of MTB from clinical specimens by PCR are in concordance with the results obtained in Tunisia using the same insertion sequence^24^ and with other studies that also documented the use of the “IS6110" for the direct diagnosis of TB with reliable results comparing to traditional techniques.^15^,^16^ The false-negative results (9 specimens were positive in culture and negative in PCR) can be ascribed either to the paucibacillary nature of the specimen, inefficient extraction of the DNA or the presence of PCR inhibitors.^28^

Moreover some of these strains could lack the insertion sequence “IS6110" as reported by El Baghdadi *et al.* in some strains isolated in Morocco.^33^

Thus, the use of PCR targeting the insertion sequence “IS6110" in the routine analysis for the detection of MTB, will be of great interest for the diagnosis of TB, especially in the critical cases, such as chronic and treatment failure or relapse cases, where the rapid diagnosis is mandatory for the initiation of chemotherapy. Furthermore, for most accuracy it could be better to make a combination between clinical, radiological, bacteriological and PCR testing to confirm the presence of the MTB for a better management of TB in Morocco.

## Figures and Tables

**Table 1:** The demographical, epidemiological information about the patients and the results of conventional and molecular techniques.

Group of patients	Number of cases	Range of age	Age median	Sex %		Sex ratio	ZN Staining		Culture		PCR	
				Male	Female		Positive	Negative	Positive	Negative	Positive	Negative
**Suspected cases of TB**	30	[15–80]	48	56.66	43.33	1.30	0	30	0	30	0	30
**New cases**	188	[15–84]	40	63.29	36.70	1.72	50	138	80	108	77	111
**Treatment Failure cases**	30	[21–80]	42	83.33	16.66	5.00	4	26	9	21	8	22
**Treatment relapse cases**	41	[19–70]	43	63.41	36.58	1.73	16	25	21	20	21	20
**Chronic cases**	16	[20–59]	41	62.50	37.50	1.66	5	11	6	10	5	11
**Total of Cases**	305	[15–84]					75	230	116	189	111	194

**Table 2:** The results of conventional examination and PCR according to the kind of specimen.

Kind of specimen	Total Number	ZN staining		Culture		PCR	
		Positive	Negative	Positive	Negative	Positive	Negative
**Pulmonary specimens**							

Sputum	274	70	204	110	164	107	167
BAL	3	-	3	1	2	1	2
Bronchic Aspiration	2	-	2	1	1	1	1
Sputum induced by Fibroscopy	1	-	1	-	1	-	1

**Extrapulmonary specimens**							

LP	21	-	21	4	17	2	19
Biopsy	1	-	1	-	1	-	1
Abscess	1	-	1	-	1	-	1
Urine	1	-	1	-	1	-	1
Gastric liquid	1	-	1	-	1	-	1

**Table 3:** Comparison of MTB detection by the direct microscopy smear and molecular techniques.

Techniques of detection of MTB		Conventional technique (Direct Examination)		Total
		Positive	Negative	
Molecular technique (Direct PCR)	Positive	68	43	111
	Negative	7	187	194
Total		75	230	305

**Table 4:** Comparison of MTB detection by culture and molecular techniques.

Techniques of detection of MTB		Conventional technique (culture)		Total
		Positive	Negative	
Molecular technique (Direct PCR)	Positive	107	4	111
	Negative	9	185	194
Total		116	189	305
